# Transplant Trial Watch

**DOI:** 10.3389/ti.2022.10905

**Published:** 2022-11-01

**Authors:** John M. O’Callaghan

**Affiliations:** ^1^ University Hospitals Coventry and Warwickshire, Coventry, United Kingdom; ^2^ Centre for Evidence in Transplantation, University of Oxford, Oxford, United Kingdom

**Keywords:** living kidney donation, systematic review, pregnancy, lung transplantation, bronchiolitis obliterans syndrome

To keep the transplantation community informed about recently published level 1 evidence in organ transplantation ESOT and the Centre for Evidence in Transplantation have developed the Transplant Trial Watch. The Transplant Trial Watch is a monthly overview of 10 new randomised controlled trials (RCTs) and systematic reviews. This page of Transplant International offers commentaries on methodological issues and clinical implications on two articles of particular interest from the CET Transplant Trial Watch monthly selection. For all high quality evidence in solid organ transplantation, visit the Transplant Library: www.transplantlibrary.com.

**Randomised Controlled Trial 1**

Pregnancy After Living Kidney Donation, A Systematic Review of the Available Evidence and a Review of the Current Guidance.

*by Pippias, M., et al. American Journal of Transplantation [online ahead of print].*

## Aims

The aim of this study was to identify all available evidence investigating pregnancy complications post-living kidney donation, and to compare the quality and consistency of guidelines focusing on pregnancy in living kidney donors.

## Interventions

A literature search was conducted on Embase, PubMed, MEDLINE, society webpages and guideline registries. Three independent reviewers performed the initial screening of study titles and abstracts. Eligibility assessment of full-text articles and data extraction were carried out by two independent reviewers. The methodological quality of the included studies were assessed using the Risk Of Bias In Non-randomized Studies of Interventions (ROBINS-I) tool.

## Participants

16 studies were included in the review.

## Outcomes

The main outcomes of interest were post-donation pregnancy complications, and the risk of adverse maternal, fetal and neonatal outcomes.

## Follow-Up

Not applicable.

## CET Conclusion

This systematic review summarises the literature and guidelines relating to pregnancy following living kidney donation. The authors identified 16 studies reporting on 1399 post-donation pregnancies. Whilst the risk of pre-eclampsia increased post-donation, it is in keeping with an unselected general population. No difference was found in risk of other pregnancy or foetal complications. Guidelines were found to be generally consistent in advice. Methodology appears good, with well-described searches across a number of databases and screening by 3 reviewers. Risk of bias was assessed with the Robins-I tool and found to be low-moderate in most studies. Of note, studies were published over a long period (35 years) so it is perhaps not clear how relevant results of early studies are to today’s practice. Overall, the authors graded the certainty of evidence in risk of hypertension and pre-eclampsia as “low” and for other foetal outcomes as “very low,” reflecting the quality and size of the underlying evidence. This paper provides a very good summary of the evidence (and limitations thereof) regarding post-donation pregnancy.

## Funding Source

Non-industry funded.

**Randomised Controlled Trial 2**

Impact of Lung Function Decline on Mortality in Lung Transplant Recipients: Long-Term Results From the L-CsA-i Study for the Prevention of Bronchiolitis Obliterans Syndrome.

*by Kneidinger, N., et al. Frontiers in Medicine 2022; 9: 897581.*

## Aims

This study aimed to determine the association between forced expiratory volume in one second (FEV1) and risk of mortality in patients following lung transplantation, using the 10-year follow up data from the PARI Study No. 12011.201.

## Interventions

Participants in the original trial were randomised to receive either liposomal Cyclosporine A inhalation (L-CsA-i) or placebo.

## Participants

130 lung transplant recipients.

## Outcomes

The main outcomes of interest were the association between the course of post-transplant FEV1 over time and the risk of mortality, time to progression to allograft dysfunction and survival.

## Follow-Up

10 years.

## CET Conclusion

This paper presents post hoc analyses from a previously published RCT. The original RCT investigated inhaled liposomal ciclosporin-A in the prevention of Bronchiolitis Obliterans Syndrome (BOS) after lung transplantation. 10-year follow up is now available for all 130 of the included patients. A strong association was found between baseline FEV1 and mortality risk and each 1% drop from baseline FEV1 was associated with 3.5% increased risk for mortality. The individual trajectories in lung function were highly variable between patients, however it seems that post-transplant FEV1 is a valid predictor of mortality and could be used to institute pre-emptive treatment.

## Trial Registration

ClinicalTrials.gov—NCT01334892.

## Funding Source

Industry funded.

## Clinical Impact Summary

This paper presents some long-term follow up from a previously published RCT of inhaled liposomal cyclosporine A in lung transplantation. The original study closed prior to reaching the target patient inclusion due to very slow accumulation of cases (1).

The paper by Kneidinger et al presents post hoc analyses from the RCT. The authors used the collected data to explore the relationship between decline in FEV1 and mortality in patients with single and double lung transplant.

Whilst patients were included in the trial they had FEV1 measurements every 2 months, and for this analysis they were requested every 6 months up to 10 years from inclusion. Complete data was retrieved for 91% of included patients, and reduced data for the remaining patients, censored at the last study visit. Mean follow up was 61 months.

On average, FEV1 deteriorated over time but the trajectory for showed a great deal of diversity between patients. A highly significant correlation was found between the relative drop in FEV1 compared to baseline and mortality. In broad terms a 1% reduction in FEV1 compared to baseline, related to 3.4% higher mortality risk. In cox regression analysis, type of transplant was the only significant independent predictor of mortality; with recipients of single lung transplants having increased risk of progression.

A significant amount of lung function must be lost before chronic lung allograft dysfunction can be diagnosed. Understanding the decline in FEV1 might allow early intervention and improvement in patient care through modification of the underlying process. Decline in FEV1 from baseline could also be used as a reliable surrogate outcome for mortality in clinical trials.
